# Retinol but not retinoic acid can enhance the glutathione level, in a manner similar to β‐carotene, in a murine cultured macrophage cell line

**DOI:** 10.1002/fsn3.726

**Published:** 2018-07-20

**Authors:** Yuuka Mukai, Rintaro Yamanishi

**Affiliations:** ^1^ Department of Food Hygiene and Function School of Nutrition and Dietetics Faculty of Health and Social Work Kanagawa University of Human Services Kanagawa Japan; ^2^ Department of Food Science and Nutrition School of Nutrition and Dietetics Faculty of Health and Social Work Kanagawa University of Human Services Kanagawa Japan

**Keywords:** β‐carotene, glutathione, RAW264 cells, retinoic acid signaling pathway, retinol

## Abstract

**Scope:**

We evaluated the potential of retinol and retinoic acid (RA) to enhance intracellular glutathione (GSH) levels in a murine cultured macrophage cell line, RAW264, to investigate whether the RA signaling pathway is involved in the β‐carotene‐induced GSH enhancement.

**Methods and results:**

We examined GSH levels in RAW264 cells cultured in media supplemented with β‐carotene and various inhibitors (ER50891 for RA receptor (RAR)α, CD2665 for RARβ/γ, or HX531 for all subtypes of retinoid X receptor (RXR)), to verify each inhibitor's activity against β‐carotene, as well as in media supplemented with various stimulants (AM80 for RARα, CD2314 for RARβ, CD437 for RARγ, or SR11237 for RXR), to compare their activity with that of β‐carotene. We also examined the GSH level and glutamate‐cysteine‐ligase (GCL) expression in RAW264 cells cultured in all‐*trans* RA‐ or retinol‐supplemented media. Enhanced GSH production was not inhibited by any tested antagonist, and, apart from β‐carotene, no agonist induced GSH production. Retinol, but not all‐*trans* RA, enhanced GSH synthesis and increased GCL expression, similar to that observed with β‐carotene.

**Conclusion:**

The RA signaling pathway may not be involved in the β‐carotene‐induced enhancement of GSH levels in RAW264 cells, whereas, like β‐carotene, retinol can enhance the GSH level and GCL expression.

## 1 INTRODUCTION

β‐Carotene, a precursor of vitamin A, is the most commonly consumed carotenoid. In the human small intestine, β‐carotene is directly absorbed as an intact molecule or as two molecules of retinal, which are generated upon hydrolysis of β‐carotene by β‐carotene 15,15′‐dioxygenase [EC 1.13.11.63]. Retinal and retinol (vitamin A) are interconvertible in the body, as needed. Retinal is oxidized into retinoic acid (RA) in the presence of retinaldehyde dehydrogenase [EC 1.2.1.36] or retinal oxidase [EC 1.2.3.11] (Patel & Vajdy, 2015). Retinol, retinal, and RA, which can be metabolites of β‐carotene, are collectively known as retinoids. We previously reported that supplementation of culture media with β‐carotene‐enhanced intracellular glutathione (GSH) levels in a murine macrophage cell line RAW264 (Imamura, Bando, & Yamanishi, 2006). GSH is a γ‐glutamyl tripeptide consisting of L‐glutamate, L‐cysteine, and L‐glycine. It is a representative intracellular antioxidative agent that can neutralize reactive oxygen species and free radicals. In addition, we demonstrated that the mRNA and protein expression of glutamate‐cysteine‐ligase (GCL) [EC 6.3.2.2], the rate‐limiting enzyme in GSH synthesis, was upregulated in RAW264 cells cultured in media supplemented with β‐carotene (Akaboshi & Yamanishi, 2014).

Retinoic acid plays a role in a wide range of biological processes mediated through binding and activation of the nuclear receptors, RA receptor (RAR), and retinoid X receptor (RXR). Three subtypes of RAR (α, β, and γ) and three subtypes of RXR (α, β, and γ) have been isolated. RARs are bound and activated by all‐*trans* RA and its 9‐*cis* isomer, while RXRs are bound and activated by the 9‐*cis*‐RA only. Heterodimers of activated RAR and RXR act as ligand‐dependent transcription factors. Numerous studies have demonstrated that the RA signaling pathway, which is mediated via RAR and/or RXR, can modulate the expression of genes involved in cell growth (Clarke, Germain, Altucci, & Gronemeyer, 2004), energy metabolism (Zhang, Wang, Li, & Chen, 2015), and immune responses (Iwata, 2009; Nagy, Szanto, Szatmari, & Szeles, 2012). However, the involvement of the RA signaling pathway in the enhancement of GSH production has not been reported in macrophages.

One aim of this study was to clarify whether the RA signaling pathway would mediate the β‐carotene‐induced enhancement of GSH production in macrophages. To this end, we examined intracellular GSH level in RAW264 cells cultured in media supplemented with β‐carotene and various retinoid receptor (RAR and RXR) antagonists. Additionally, we examined the intracellular GSH level in RAW264 cells cultured in media supplemented with various retinoid receptor agonists to compare their activities on GSH production with that of β‐carotene. Additionally, We also aimed to clarify whether retinol and/or RA, as well as β‐carotene, could enhance GSH production and expression of its rate‐limiting enzyme, GCL, in macrophages. To this end, we examined the intracellular GSH level and the level of the modulator subunit of GCL (GCLm) in RAW264 cells cultured in media supplemented with retinol, RA, or β‐carotene. Then, we discuss the involvement or noninvolvement of the RA signaling pathway in the β‐carotene‐induced increase in GSH production in RAW264 cells and the activity of retinoids on the increase in GSH production.

## 2 MATERIALS AND METHODS

### 2.1 Chemicals

Nonessential amino acids, penicillin‐streptomycin, and L‐glutamine were purchased from Life Technologies (Carlsbad, CA, USA). Fetal bovine serum was purchased from Moregate (Bulimba, Australia). Minimum essential medium and β‐carotene were purchased from Sigma‐Aldrich (St. Louis, MO, USA). Trypsin‐EDTA, DMSO, Nonidet P‐40, and BSA F‐V were purchased from Nacalai Tesque (Kyoto, Japan). THF was purchased from Kanto Chemical (Tokyo, Japan). All‐*trans* RA, GSH, and 5,5′‐dithiobis(2‐nitrobenzoic acid) (DTNB) were purchased from Wako Pure Chemical Industries (Osaka, Japan). Glutathione reductase and nicotinamide adenine dinucleotide phosphate reduced form (NADPH) were purchased from Oriental Yeast (Tokyo, Japan). 4‐[5‐[8‐(1‐Methylethyl)‐4‐phenyl‐2‐quinolinyl]‐1H‐2‐pyrrolyl]‐benzoic acid (ER50891, RARα antagonist), 4‐[6‐[(2‐Methoxyethoxy)methoxy]‐7‐tricyclo[3.3.1.13,7]dec‐1‐yl‐2‐naphthalenyl]benzoic acid (CD2665, RARβ/γ antagonist), 4‐(7,8,9,10‐Tetrahydro‐5,7,7,10.10‐pentamethyl‐2‐nitro‐5H‐benzo[b]naphtho[2,3‐e][1,4]‐diazepin‐12‐yl)‐benzoic acid (HX531, RXR antagonist), 4‐[[(5,6,7,8‐Tetrahydro‐5,5,8,8‐tetramethyl‐2‐naphthalenyl)amino]carbonyl]benzoic acid (AM80, RARα agonist), 5‐(5,6,7,8‐Tetrahydro‐5,5,8,8‐tetramethyl‐2‐anthracenyl)‐3‐thiophenecarboxylic acid (CD2314, RARβ agonist), 6‐(4‐Hydroxy‐3‐tricyclo[3.3.1.13,7]dec‐1‐ylphenyl)‐2‐naphthalenecarboxylic acid (CD437, RARγ agonist), and 4‐[2‐(5,6,7,8‐Tetrahydro‐5,5,8,8‐tetramethyl‐2‐naphthalenyl)‐1,3‐dioxolan‐2‐yl]‐benzoic acid (SR11237, RXR agonist) were purchased from Tocris Bioscience (Bristol, UK). The anti‐GCLm antibody, γ‐GCSm F‐8, was purchased from Santa Cruz Biotechnology (Santa Cruz, CA, USA). Anti‐β‐actin mAb, B11V08, was purchased from BioVision (Mountain View, CA, USA). Peroxidase‐conjugated goat anti‐mouse IgG was purchased from Sigma‐Aldrich. ECL reagents for western blotting were purchased from GE Healthcare UK (Buckinghamshire, UK).

### 2.2 Preparation of culture medium

β‐Carotene, retinol, and all‐*trans* RA were dissolved in THF (final concentration in a medium: 0.2%), as a vehicle, to prepare a stock solution, which was then stored in the dark at −80°C under N_2_ atmosphere. ER50891, CD2665, HX531, AM80, CD2314, CD437, and SR11237 were dissolved in DMSO (final concentration in a medium: 0.1%), as a vehicle, to prepare a stock solution, which was then stored at −20°C. Each stock solution was then added to a standard medium consisting of minimum essential medium supplemented with 10% fetal bovine serum, L‐glutamine, nonessential amino acids, and 50,000 U/L penicillin‐streptomycin. Solvents without test materials were added to the standard medium for the preparation of control medium. Medium containing each test material was prepared just before its use because the materials were not very stable in the medium.

To evaluate the effects of retinoid receptor antagonists on intracellular GSH levels, the test medium contained 20 μM β‐carotene and 2.5, 10, or 25 μM (if possible in solubility) of ER50891, CD2665, or HX531. These concentrations of retinoid receptor antagonists have been reported in several studies, which demonstrated the inhibitory effect of the antagonists on RAR (α, β, and γ) and RXR, using culture cell lines (Kim, Ciletti, Michel, Reichert, & Rosenfield, 2000; Somenzi et al., 2007; Suzuki et al., 2009).

To evaluate the effects of retinoid receptor agonists on intracellular GSH levels, the test medium contained 0.001, 0.1, or 10 μM of AM80, CD2314, CD437, or SR11237. These concentrations of retinoid receptor agonists have been reported in several studies, which demonstrated the ligand activity of the agonists on RAR (α, β, and γ) and RXR, using culture cell lines (Di Lascio et al., 2016; Duprey‐Diaz, Blagburn, & Blanco, 2016; Gendimenico, Stim, Corbo, Janssen, & Mezick, 1994; Jimi et al., 2007; Zhao & Spanjaard, 2003).

### 2.3 Cell culture

RAW264 cells were provided by the RIKEN BRC through the National Bio‐Resource Project of the MEXT/AMED, Japan. The cells were grown in standard medium in a humidified atmosphere of 5% CO_2_ and 95% air at 37°C. For experiments, 1.2–1.5 × 10^6^ cells/well were cultured with 3 ml of test media supplemented with various concentrations of test materials for 15 hr in 6‐well plastic plates. Harvested whole cells from each well were analyzed to measure intracellular GSH levels and protein content.

### 2.4 Quantification of total intracellular GSH

After culturing in test media supplemented with various concentrations of test materials, cells were harvested, washed with PBS, sonicated in 0.5% Nonidet P‐40, and centrifuged at 8,000 × *g* for 10 min at 4°C. The supernatant was then collected. Intracellular GSH levels were determined by the DTNB‐glutathione reductase recycling assay after protein precipitation with 5% sulfosalicylic acid, as described previously (Anderson, 1985). In brief, GSH is oxidized by DTNB to give 5‐mercapto‐2‐nitrobenzoic acid and glutathione disulfide, and the glutathione disulfide is reduced to GSH by the action of glutathione reductase [EC 1.8.1.7] and NADPH. The rate of 5‐mercapto‐2‐nitrobenzoic acid formation is recorded at 405 nm to determine the total GSH level, which is expressed as the sum of GSH and glutathione disulfide. The total intracellular GSH level was normalized to the protein concentration in each well, which was measured with a Protein Assay Bicinchoninate kit (Nacalai Tesque), using BSA F‐V as a protein standard. The phrase “GSH level” refers to the total intracellular GSH level normalized to the intracellular protein concentration.

### 2.5 Western blotting

Harvested cells were solubilized in lysis buffer (20 mM Tris‐HCl, pH 7.6, 0.137 M NaCl, 10% glycerol, 1 mM PMSF, and 1% Nonidet P‐40) containing a protease inhibitor cocktail (Roche Diagnostics Japan, Tokyo, Japan) with sonication, and centrifuged to remove precipitates. Cell lysates were separated by SDS‐PAGE on a 10% polyacrylamide gel. Proteins were then electrophoretically transferred onto a polyvinylidene difluoride membrane using the iBlot^™^2 Dry Blotting System (Thermo Fisher Scientific, Waltham, MA, USA). The membrane was immunostained with anti‐GCLm or anti‐β‐actin, as a primary antibody, and peroxidase‐conjugated goat anti‐mouse IgG, as a secondary antibody, using the iBind^™^ Western System (Thermo Fisher Scientific) at room temperature according to the manufacturer's instructions. The immunocomplexes on the membrane were detected with ECL reagents according to the manufacturer's instructions. Images were obtained using an ATTO cooled CCD camera system Ez‐Capture (ATTO Corp., Tokyo, Japan). Relative protein expression of GCLm was normalized against β‐actin expression as a reference protein. We preferentially measured the enhancement of GCLm protein expression in this study because it had been more evident than that of the catalytic subunit of GCL (Akaboshi & Yamanishi, 2014).

### 2.6 Statistical analysis

Statistical analyses were performed using IBM SPSS Statistics version 23 (IBM Corp., Somers, NY, USA). Data are expressed as the mean ± *SD* of triplicate samples. Statistical significance was determined by one‐way analysis of variance and Bonferroni multiple comparisons test. *p *<* *0.05 was considered statistically significant.

## 3 RESULTS

**Figure 1:**
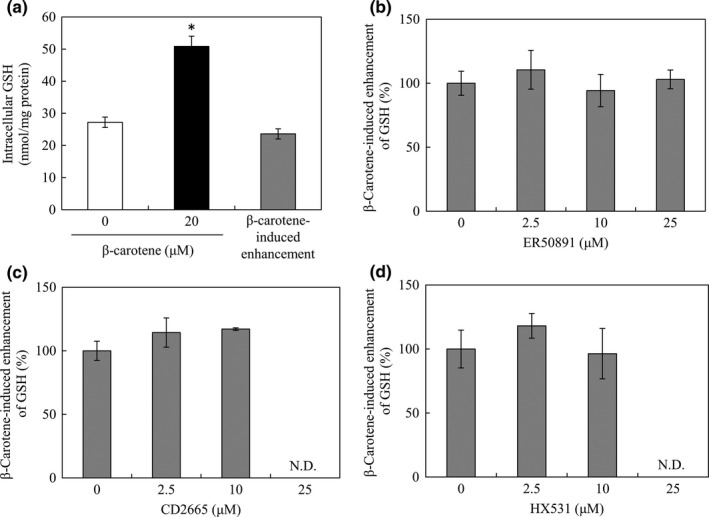
Effects of RAR‐ and RXR‐specific antagonists on the β‐carotene‐induced enhancement of intracellular glutathione (GSH) level in RAW264 cells. RAW264 cells were cultured in media supplemented with β‐carotene only (a), or β‐carotene and different concentrations of the retinoid receptor antagonists, ER50891 (b), CD2665 (c), or HX531 (d). RAW264 cells (1.5 × 10^6^) were cultured in 6‐well plastic plates in a medium containing 2.5, 10, or 25 μM of each antagonist with or without 20 μM β‐carotene and incubated at 37°C for 15 hr. Harvested cells were used to quantify GSH. In panel a, the white bar indicates the intracellular GSH level without β‐carotene, the black bar indicates intracellular GSH levels with β‐carotene, and the gray bar indicates the β‐carotene‐induced enhancement of GSH, calculated from the first two values. In panel b~d, increased ratio of GSH was calculated by dividing the β‐carotene‐induced enhancement of GSH concentration in cells cultured in media with antagonists by that in control cells and expressed as a percentage, after normalization of the intracellular GSH level to the protein concentration of each well. Data are expressed as mean ± *SD* of triplicate samples. *p <* *0.01, compared with the control cells. N.D. refers to not determined

As previously described (Imamura et al., 2006; Katsuura, Imamura, Bando, & Yamanishi, 2009), the GSH level in RAW264 cells cultured with β‐carotene was significantly increased compared to that in cells cultured in a medium without β‐carotene (Figure 1a). To examine the involvement of the stimuli via RAR or RXR there, the inhibitory effects of RAR‐ or RXR‐specific antagonists on the β‐carotene‐induced enhancement of intracellular GSH levels were investigated. By dividing by a datum with no antagonist, the ratio of the residual β‐carotene‐induced enhancement of GSH level in cells cultured with antagonist was calculated (Figure 1b–d). When cells were cultured in media supplemented with any retinoid receptor antagonist, i.e., ER50891 for RARα, CD2665 for RAR β and γ, or HX531 for all RXR subtypes, the β‐carotene‐induced enhancement of GSH level did not show any significant decrease. The data for 25 μM CD2665 are not presented owing to its toxicity, as judged from the amount of recovered protein. The data for 25 μM HX531 are not presented either owing to a solubility problem with the antagonist.

**Figure 2:**
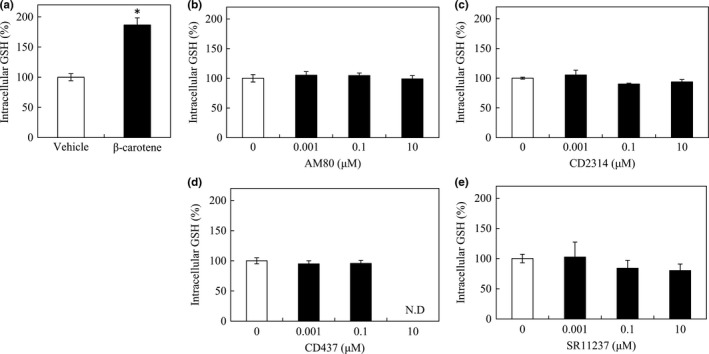
Effects of RAR‐ and RXR‐specific agonists on the intracellular glutathione (GSH) level in RAW264 cells. RAW264 cells were cultured in media supplemented with 20 μM β‐carotene (a), or different concentrations of the retinoid receptor agonists, AM80 (b), CD2314 (c), CD437 (d), or SR11237 (e). RAW264 cells (1.5 × 10^6^) were cultured in 6‐well plastic plates in a medium containing 0.001, 0.1, or 10 μM of each agonist and incubated at 37°C for 15 hr. Harvested cells were used to quantify GSH. The relative GSH levels were quantified as a percentage of that in control cells with vehicle (THF in panel a and DMSO in panel b~e) only, after normalization to the protein concentration in each well. Data are expressed as mean ± *SD* of triplicate samples. *p <* *0.01, compared with the control cells. N.D. refers to not determined

RAW264 cells were treated with several representative RAR‐ or RXR‐specific agonists. When RAW264 cells were cultured in media supplemented with any specific synthetic agonists for various RAR and RXR subtypes, i.e., AM80 for RARα, CD2314 for RARβ, CD437 for RARγ, and SR11237 for all RXR subtypes, the GSH level in RAW264 cells was not significantly increased at any of the concentrations tested (Figure 2b–e). The data for 10 μM CD437 are not presented owing to its toxicity, similar to that of 25 μM CD2665 described above.

**Figure 3:**
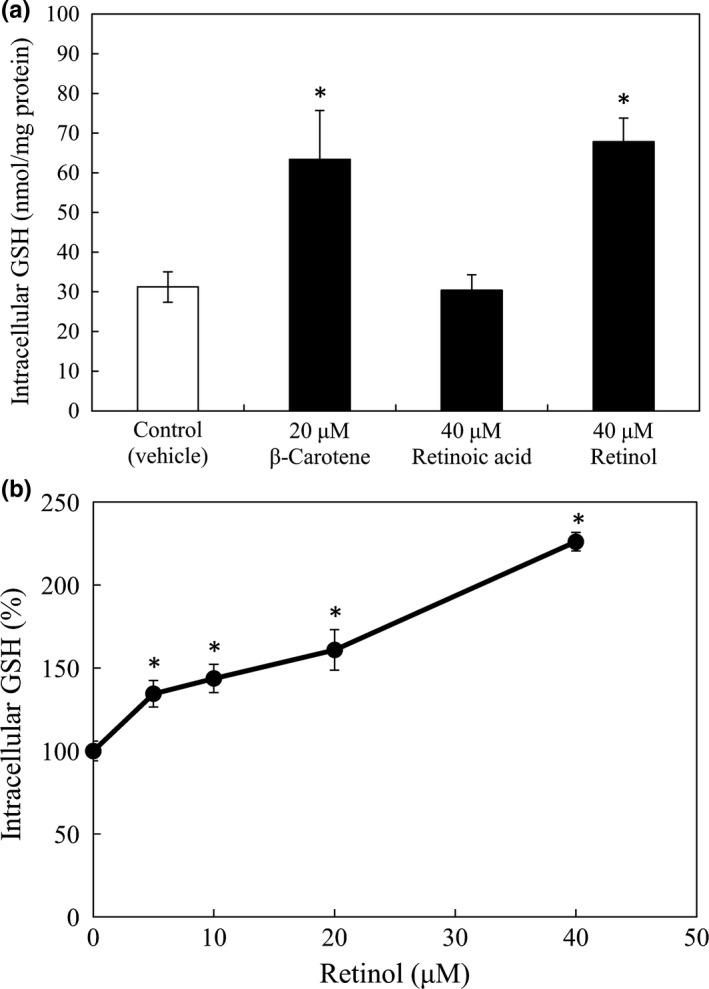
Intracellular glutathione (GSH) level in RAW264 cells cultured in media supplemented with retinoids. RAW264 cells (1.5 × 10^6^) were cultured in 6‐well plastic plates in medium containing 20 μM β‐carotene, 40 μM retinoic acid (RA), or 40 μM retinol (panel a), and 5, 10, 20, or 40 μM retinol (panel b) at 37°C for 15 hr. Harvested cells were used to quantify GSH. The relative GSH levels were quantified as a percentage of that in control cells with vehicle (THF) only, after normalization of the intracellular GSH level to the protein concentrations of each well. Data are expressed as mean ± *SD* of triplicate samples. *p <* *0.01, compared with the control cells

To determine whether retinoids, which are the metabolites of β‐carotene, can enhance the intracellular GSH level, RAW264 cells were treated with 20 μM β‐carotene, 40 μM all‐*trans* RA, or 40 μM retinol because two molecules of retinoids can be generated from one molecule of β‐carotene. Retinol, but not all‐*trans* RA, exhibited an ability comparable to that of β‐carotene to enhance the intracellular GSH level (Figure 3a). When RAW264 cells were cultured in a medium supplemented with 5, 10, 20, or 40 μM retinol, the intracellular GSH level increased in a dose‐dependent manner (Figure 3b).

**Figure 4:**
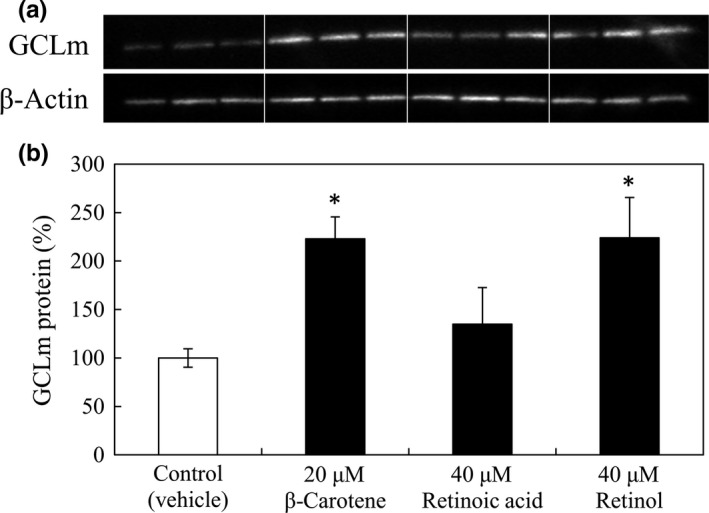
Glutamate‐cysteine‐ligase, modulator subunit (GCLm) protein expression in RAW264 cells cultured in media supplemented with β‐carotene, RA, or retinol. In panel a, the protein expression of GCLm was detected by western blotting. In addition to the images, the calculated relative protein expression of the GCLm is presented in panel b. RAW264 cells (1.5 × 10^6^) were cultured in 6‐well plastic plates in a medium containing 20 μM β‐carotene, 40 μM RA, or 40 μM retinol at 37°C for 15 hr. Harvested cells were used for western blotting to detect GCLm or β‐actin. The relative GCLm protein expression in panel b was quantified as a percentage of that in control cells with vehicle (THF) only, shown as the white bar, after normalization of the β‐actin protein expression in each sample. Data are expressed as mean ± *SD* of triplicate samples. *p <* *0.01, compared with the control cells

We have previously reported that the β‐carotene‐induced increment of the intracellular GSH level resulted from the enhancement of GCLm protein expression (Akaboshi & Yamanishi, 2014). To examine if retinol can induce an increase in the GCLm protein level similar to that induced by β‐carotene, the protein levels in RAW264 cells cultured in a medium supplemented with β‐carotene, retinol, or RA were compared by western blotting. When incubated with 40 μM retinol, the GCL protein level in RAW264 cells was significantly higher than that of the control, which was comparable to that observed with 20 μM β‐carotene (Figure 4). On the other hand, the GCL protein level in RAW264 cells was not influenced by 40 μM all‐*trans* RA, which also did not increase the GSH level (Figure 3a).

## 4 DISCUSSION

As β‐carotene can be metabolized to retinoids including RA, which may mediate a wide range of biological processes, it was necessary to determine whether the RA signaling pathway would be involved in the β‐carotene‐induced enhancement of GSH production in RAW264 macrophages. The major findings of the present study are as follows: (a) the β‐carotene‐induced enhancement of GSH level may not be regulated by the RA signaling pathway via RAR and/or RXR; and (b) retinol could enhance both GSH production and the protein expression of GCL, in RAW264 macrophages, whereas these parameters were not affected by all‐*trans* RA.

In this study, we found that in RAW264 macrophages, none of the tested antagonists against RAR and RXR, i.e., ER50891, CD2665, and HX531 could inhibit the β‐carotene‐induced enhancement of GSH production (Figure 1), and none of the agonists for RAR and RXR, i.e., AM80, CD2314, CD437, and SR11237 could induce the enhancement of GSH production as β‐carotene could (Figure 2). These results indicated that the RA signaling pathway might not be involved in the increase in GSH production triggered by β‐carotene in RAW264 macrophages. Enhancing the GSH levels, a possible novel role of β‐carotene in physiology, may be mediated via mechanisms independent of the RA signaling pathway.

We also found that retinol could increase the intracellular level of GSH (Figure 3) and the GCLm protein (Figure 4), in RAW264 macrophages, whereas all‐*trans* RA was not able to enhance either of them. Since GCL is the rate‐limiting enzyme in GSH synthesis, the upregulation of GCL expression by retinol may account for the enhancement of GSH synthesis by retinol in the macrophages.

We have demonstrated previously that β‐carotene induced the enhancement of GSH production and GCL protein expression in a c‐Jun N‐terminal kinases (JNK) pathway‐related manner (Akaboshi & Yamanishi, 2014). JNK is one of the subfamilies of mitogen‐activated protein kinases, which is involved in redox‐related cellular functions such as cell cycle progression and cell survival or death responses (Son, Kim, Chung, & Pae, 2013). The involvement of the JNK pathway in the β‐carotene‐induced enhancement of GSH level was evidenced by suppressed expression of the GCL protein by a JNK inhibitor and enhanced JNK phosphorylation by β‐carotene (Akaboshi & Yamanishi, 2014). However, there are a number of studies that have demonstrated that JNK activation suppresses RA signaling through RARα degradation or phosphorylation (Hoshikawa et al., 2011; Singh, Guleria, Nizamutdinova, Baker, & Pan, 2012; Srinivas et al., 2005). Therefore, the results of the present study, which indicate the existence of a mechanism independent of the RA signaling pathway in the β‐carotene and retinol‐induced enhancement of GSH level in RAW264 cells, can be consistent with the involvement of JNK pathway.

In this study, it remains unclear which is the exact molecule that induced the enhancement of GSH production in RAW264 cells. However, one possibility is that β‐carotene is metabolized into retinol, and then retinol actually exerts the effect on enhancement of GSH synthesis. It is well‐known that β‐carotene is pro‐vitamin A, and that retinol (vitamin A) converted from β‐carotene has potential for physiological functions, the representative one of which is the sense of vision. It was reported that retinol was not detected in RAW264 cells after incubation with culture medium supplemented with β‐carotene in the previous study (Katsuura et al., 2009). Likewise, it was also reported there that the mRNA for β‐carotene‐15,15′‐monooxygenase (BCMO1), which catalyzes the production of retinoids from β‐carotene or β‐cryptoxanthin, was not detected in RAW264 cells. In contradiction to that, Zolberg et al. reported that BCMO1 protein and its product retinol were detected in RAW264.7 cells after the incubation with 9‐cis β‐carotene (Zolberg Relevy et al., 2015). Another possibility is that both β‐carotene and retinol may, at least in part, have almost the same effect on the enhancement of GSH synthesis in RAW264 cells. Retinol is known to have a redox‐sensitive characteristic of a hydrophobic isoprenoid (Noy, 2000). These chemical properties of retinol are similar to those of β‐carotene. Therefore, it is possible that the structural kinship of both molecules may be related to their functional kinship on redox‐status via regulation of GSH synthesis in macrophages. In order to elucidate these points, further investigation will be needed. In addition, in this experiment, we used much higher concentration of β‐carotene and retinol in vitro than their physiological levels. Thus, it is still uncertain that β‐carotene and retinol can directly upregulate GCLm expression and concomitant GSH production in vivo.

In conclusion, we herein demonstrated that the RA signaling pathway mediated via RAR and/or RXR may not be involved in the β‐carotene‐induced enhancement of GSH level in RAW264 cells. We also found that retinol, but not all‐*trans* RA, enhanced the synthesis of GSH, accompanied by increased protein expression of GCL in RAW264 cells, similar to that observed with β‐carotene. Although these observations were made in vitro, these findings may give us a new aspect for the physiological function of vitamin A‐related compounds, i.e., retinol and β‐carotene.

## CONFLICT OF INTEREST

The authors have declared no conflicts of interest.

## AUTHOR CONTRIBUTIONS

R.Y. designed and conducted the study. Y.M. analyzed the data and performed statistical analysis. Y.M. wrote the manuscript.

## ETHICAL STATEMENTS

This study does not involve any human and animal testing.
